# Fatigue Behavior of Concrete Beams Prestressed with Partially Bonded CFRP Bars Subjected to Cyclic Loads

**DOI:** 10.3390/ma12203352

**Published:** 2019-10-15

**Authors:** Yoseok Jeong, WooSeok Kim, Viktor Gribniak, David Hui

**Affiliations:** 1 Department of Civil Engineering, Chungnam National University, Daejeon 34134, Korea; yosoksi@gmail.com; 2 Department Institute of Building Materials, Vilnius Gediminas Technical University, 10221 Vilnius, Lithuania; viktor.gribniak@vgtu.lt; 3 Department of Mechanical Engineering, University of New Orleans, New Orleans, LA 70148, USA; dhui@uno.edu

**Keywords:** carbon fiber reinforced polymer, CFRP, ductility, fatigue, partially bonded bars

## Abstract

The lack of ductility is the greatest concern in the applications of carbon fiber reinforced polymer (CFRP) materials, when used as pre-stressing reinforcements. To improve the ductility, a partially bonded FRP system which is intentionally unbonded in the middle part of the beam and bonded in both end parts of the beam has been developed and applied to prestressed concrete beams. While, many researchers investigated the instantaneous performance of partially bonded CFRP prestressed concrete beams, this study intended to evaluate the fatigue performance, the static load-carrying capacity after fatigue loading and ductility. Based on the fatigue loading tests followed by static loading tests, over-reinforced and web-confined partially bonded CFRP prestressed concrete beams exhibited satisfactory fatigue performance without cracks and stiffness degradation during fatigue loading. In addition, no degradation of load-carrying capacity was observed in static loading tests after the fatigue tests. The ductility index of concrete beams, prestressed with partially bonded CFRP bars, is combined with over-reinforcements and web-confinements, similar to that of beams prestressed with steel bars.

## 1. Introduction

Conventional steel tendons in prestressed concrete structures are naturally vulnerable to corrosion, which is exacerbated over time and may result in a decrease in structural stiffness, strength, and service life. The best solution to the corrosion problem is to use non-metallic reinforcements, such as fiber reinforced polymer (FRP) composites, which have high tensile strength and are non-corrosive, non-magnetic, and lightweight [1,2,3,4]. The FRP composites have been proposed as alternatives to steel tendons in prestressed concrete structures to overcome such deterioration problems. However, FRP tendons and concrete are both brittle materials. Therefore, concrete members that have been prestressed with FRP tendons have a tendency to fail due to brittleness. This indicates that classical ductility, which requires plastic deformation, is difficult to obtain. As a consequence, great interest has been focused on ductility when using FRP tendons in prestressed concrete structures by a large number of researchers and engineers [2,5,6,7,8,9,10,11,12].

To avoid failure due to brittleness, the prestressing reinforcement ratio should be designed to be greater than the balanced ratio, at which the rupture of FRP tendons and concrete crushing failures take place simultaneously [13]. However, the use of a high prestressing reinforcement ratio is not economical. An alternative to prevent or delay tendon rupture is to use unbonded tendons [2,7]. When FRP tendons are fully unbonded, the stresses in fully unbonded tendons are independent of the cross-section, but dependent only on the deformation of the member. Strains in the fully unbonded tendons are released from critical sections and averaged out along the beam length, which implies that fully unbonded tendons could be beneficial in improving the ductility of concrete structures [2,5,8,9,11]. Thus, although a concrete beam is prestressed with fully unbonded FRP tendons, with its prestressing reinforcement ratio being less than the balanced ratio in the bonded case, concrete compression crushing may precede the tensile rupture of FRP tendons at the ultimate stage. An unbonded FRP tendon system, consequently, could be beneficial in overcoming uneconomical over-reinforcement of FRP tendons, as well as enhancing the ductility of the beam.

However, the anchorage in a fully unbonded FRP system is a critical issue as it ensures reliable force transfer and interaction with the rest of the structure. There are no efficient and competitive prestressing systems for FRP tendons despite research over the last two decades [14,15]. Although, numerous anchor systems have been developed in the past to create different types of FRP tendons, the fundamental types of anchor methods can be classified as: Bond-type and wedge-type anchors [14]. Other types of anchors can be created using a combination of such basic anchor methods. However, these types of anchorage system, used for prestressing FRP tendons, have some drawbacks [14,15]. The application of the bond-type anchor is limited with regards to large-diameter FRP tendons due to its limited anchor force by bonding between the outer socket and FRP tendons. Under cyclic loads, the fatigue damage induced by shear deformation in the adhesive can occur earlier than the fatigue limit of the FRP, which restricts the application of the bonding anchor in fatigue-load-dominant structures. Wedge-type anchors use compression forces in order to grip the FRP tendons. The structure of FRP bars composed of longitudinally oriented filaments makes them efficient only for transferring tensile stresses. High localized stresses can be generated in the FRP tendons, which lead to the damage of fibers, and further rupturing the FRP tendons. Although many researchers [16,17,18,19] have stated that the resistance of the anchorage systems is adequate, premature slips and failure of FRP tendons have been identified by the American Concrete Institute (ACI) Committee 440 [13] as an inherent feature of such systems. To overcome these problems, the concept of a partially bonded FRP system was introduced. Lees and Burgoyne [20] first proposed partially bonded and discrete-bonded prestressed FRP tendons for concrete beams as a new reinforcing system. A partially bonded FRP tendon is similar to the fully bonded system, except that a portion of the FRP length is intentionally unbonded. The partially bonded FRP system alters the nature of how beams fail when compared to fully bonded FPR system. The experimental results showed that the ductility of the partially bonded system improved when a high load carrying capacity was sustained, in comparison to the fully bonded system [20].

The majority of investigations relating to partially bonded systems have focused on the analysis of short-term performance and ductility [10,21,22,23]. Chahrour and Soudki [21] reported that partially bonded CFRP reinforcements performed better than a fully bonded system of CFRP reinforcements, in terms of the load carrying capacity and ductility, which revealed that end-anchored, partially bonded CFRP reinforcements significantly enhanced the ultimate capacity, compared to that of a fully bonded system. Choi et al. [22] experimentally investigated the effects of various unbonded lengths of a partially bonded FRP reinforcement on ductility. It was found that ductility increased together with unbonded length, since the internal slip of the FRP bar and gradual concrete failure occurred near the ultimate stage, which led to the nonlinear behavior of the beams. Information on the short-term behavior of RC beams, prestressed with FRP reinforcement, is relatively abundant [6,10,21,23,24,25,26]. However, there is a lack of research investigating fatigue performance of concrete beam prestressed with partially bonded FRP tendons. For the general acceptance and wide-range use of FRP in civil engineering applications, the fatigue behavior of partially bonded FRP prestressed structures should be investigated.

In this current study, a partially bonded system was applied to prestressed concrete beams with FRP tendons to investigate fatigue behavior and capacity, as well as ductility enhancement in terms of ductility index (DI). An experimental program was carried out, and the test results were also discussed. Six simply supported, 3000 mm long concrete beams were fabricated with various bonding lengths of CFRP bars, the reinforcement ratio of prestressing bars, and web-confinement. Three beams were tested under fatigue loading (completing one million cycles); then, the beams were statically loaded up to failure, in order to evaluate the effect of fatigue loadings on the remaining load-carrying capacity. The other three beams were tested in static conditions up to failure for comparison purposes.

## 2. Research Significance

Since FRP bars and concrete are both brittle materials, the fracture characteristics of concrete beams, prestressed with FRP bars, are of interest because of its brittle failure. Therefore, a partially bonded system was developed to overcome brittle problems based on many investigations. The results showed that ductility of the partially bonded system had improved. In spite of the considerable amounts of results on the ductility of the concrete beam, prestressed with partially bonded FRP bars, most investigations were focused on the analysis of short-term performance and ductility. This paper generates important experimental data to address fatigue performance as well as ductility.3. Experimental program

### 2.1. Test Specimens and Jacking Setup

**Figure 1:**
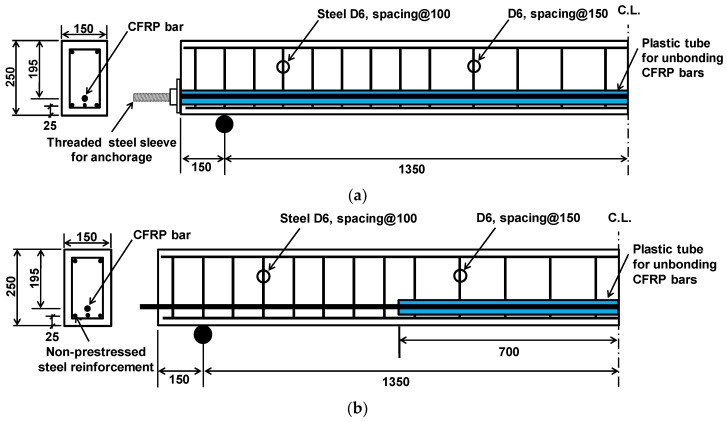

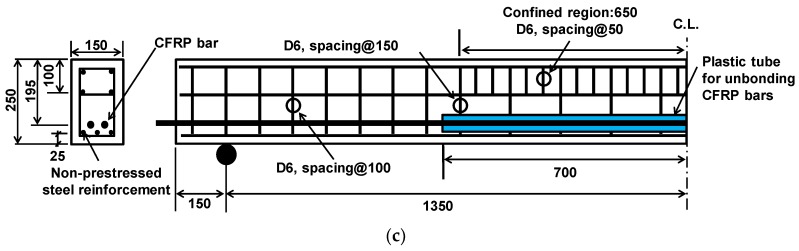
Details of beam specimens, dimension in millimeters: (**a**) F1FU/S1FU, (**b**) F1PU/S1PU, and (**c**) F2PC/S2PC (all dimensions in mm).

Six simply supported concrete beams prestressed by CFRP bars with different bonding conditions: Fully unbonded, partially bonded and fully unbonded. The main variables were the reinforcement ratio of prestressing bars, and web-confinement, including bonding conditions. The specimen identification scheme in Table 1 consists of a four-part code, A-B-C-D, where A represents the test type (F for the fatigue test or S for static test); B represents the number of CFRP bars; C represents the bonding condition (F for full unbonding or P for partial bonding), and D represents web-confinement (U for un-confinement or C for confinement). Web-confinement was introduced as a means of transverse steel closed stirrups as shown in Figure 1c. All beams were provided with minimum sectional area of the non-prestressed steel reinforcement at the bottom of the beam in accordance with ACI 318 code [27]. The minimum area has to be *A_s,min_* = 0.004*A_t_*, where *A_t_* is a part of the cross section between the flexural tension face and the center of gravity of the gross section. As non-prestressed steel bars of 6.0 mm diameter were placed below CFRP bars and in the top compressive zone with 25 mm cover. These bars also facilitate easy assembly of a reinforcement cage. The experimental program includes three sets of beams. Figure 1 and Table 1 illustrate details of the specimens.

**Figure 2:**
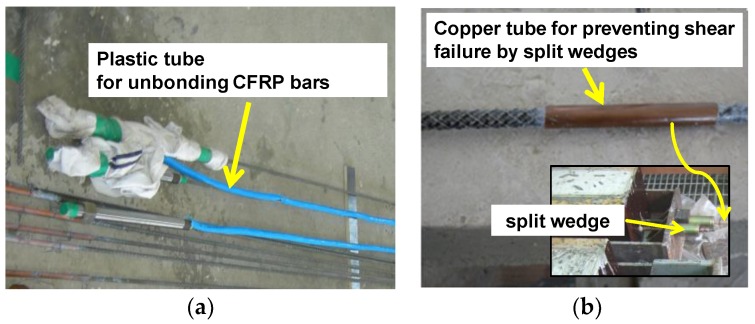
(**a**) Unbonded carbon fiber reinforced polymer (CFRP) bars and (**b**) Metal over-layered CFRP bar.

The F1FU/S1FU specimen was post-tensioned with a fully unbonded CFRP bar and no transverse confinement. A thin plastic tube was used to create the unbonded region, as shown in Figure 2a. The anchorage system for the fully unbonded FRP system is a threaded steel sleeve anchorage, by which CFRP bars are embedded with epoxy that fills tubular steel housing. The prestressing reinforcement ratios was set to 60% greater than the balanced ratio *ρ_b_*, where *ρ_b_* is the balanced ratio, the reinforcement ratio that led to simultaneous achievement of the ultimate strain of FRP bar and the ultimate compressive strain of concrete [13]. The balanced prestressing ratio for prestressed concrete beams with bonded bars can be given by [13],

(1) $$\rho_{b}=0.85\beta_{1}\frac{f\prime_{c}}{f_{pu}}\frac{\varepsilon_{cu}}{\varepsilon_{cu}+\varepsilon_{pu}-\varepsilon_{pe}}-\frac{A_{s}}{bd}\frac{f_{bu}}{f_{pu}}$$

where *β_1_* = 0.85 for concrete strengths up to 27.5 MPa, after which it is reduced at a rate of 0.05 for each 6.9 MPa of strength in excess of 27.5 MPa to a minimum value of 0.65; *f’_c_* is compressive strength of concrete, *f_pu_* is the ultimate tensile strength of the CFRP bar, *ε_cu_* is the ultimate strain in concrete in compression (= 0.003), *ε_pu_* is the ultimate tensile strain of the CFRP bar, and *ε_pe_* is the effective strain in prestressed CFRP bar, *A_s_* is total sectional area of non-prestressed reinforcement, *b* is beam width, *d* is depth to the CFRP bar (distance from extreme compression fiber to centroid of CFRP bars), *f_bu_* is the ultimate tensile strength of non-prestressed reinforcement

The F1PU/S1PU specimen has the same layout of reinforcement as the F1FU/S1FU specimen. However, unlike F1FU/S1FU, a CFRP bar was partially bonded to concrete. For the partially bonded beams, a thin plastic tube was used to make the unbonded region, whose length is 1400 mm in the mid-span as shown in Figure 2. The F1PU/S1PU specimen was pre-tensioned with a prestressing ratio of 1.6*ρ_b_*.

The F2PC/S2PC specimen has two CFRP bars partially bonded to concrete. The F2PC/S2PC specimen was over-reinforced with a prestressing reinforcement ratio of 3.2*ρ_b_*. Web-confinement was also provided with closed steel stirrups at every 50 mm as shown in Figure 1c. Conventional split wedge anchors generally for steel strands was used for pre-tensioning CFRP bars. The split wedge is not effective to grip the bare CFRP bar due to vulnerability of CFRP bar to shear stress. Therefore, CFRP bars were over-layered with metal where the split wedge was placed for gripping CFRP bars. Metal over-layered bars were fabricated by covering bars with a soft metal such as a copper, as shown in Figure 2b.

**Figure 3:**
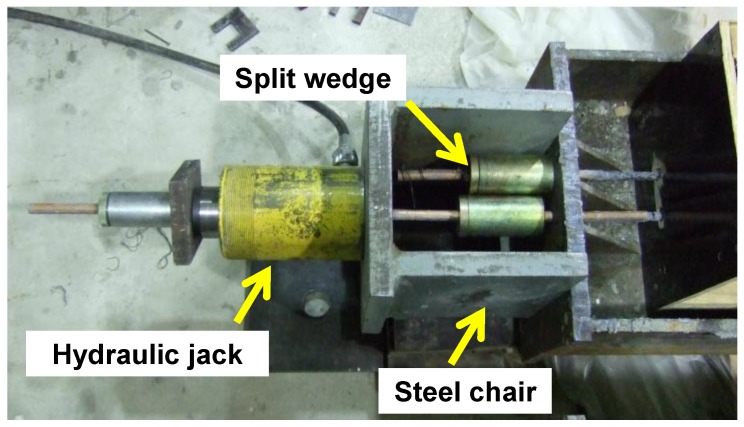
Jacking setup.

The prestressing system consisted of a 200 kN hydraulic jack (Enerpac, Menomonee Falls,WI, USA), a pressure dial gage (Enerpac, Menomonee Falls, WI, USA), and a prestressing steel chair (see Figure 3). The jacking force was monitored using both the dial gage (Enerpac, Menomonee Falls, WI, USA) and strain gages (Tokyo Measuring Instruments Lab., Tokyo, Japan) attached to CFRP bars. Elongation of the bar was also monitored at every load increment.

The beam specimens were cast in one batch. The CFRP bar has a rough surface pattern with double helical rib geometry for mechanical interlock (see Figure 2b). The average 28-day compressive strength of the concrete, according to three cylinder tests [28] was 35 MPa. Table 2 summarizes the mechanical properties of CFRP bars, and non-prestressing steel reinforcement, whose properties were provided by the manufacturer.

### 2.2. Test Program

**Figure 4:**
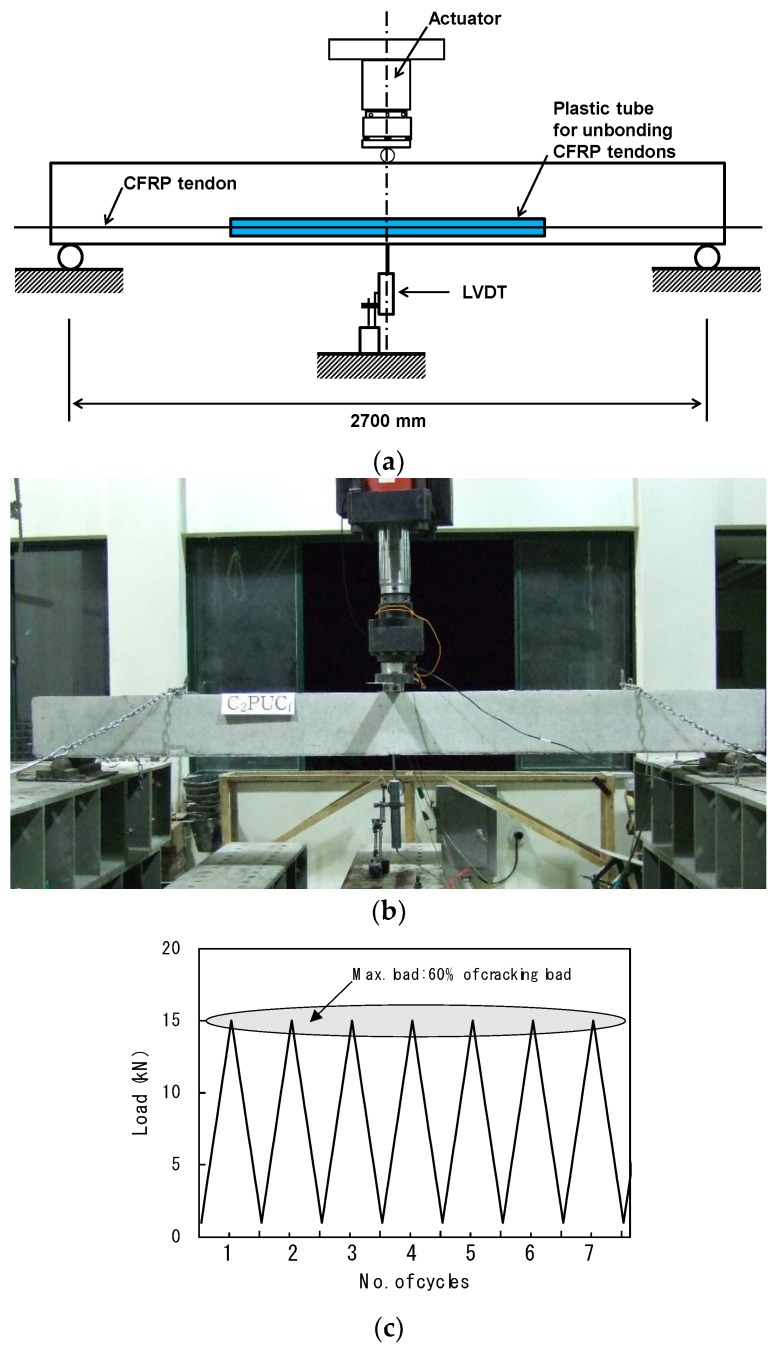
Loading setup: (**a**) loading scheme dimensions in millimeters, (**b**) photograph, and (**c**) cyclic loading layout.

Three-point bending fatigue tests were carried out. Three beams with span 2700 mm were loaded, as shown in Figure 4. The beams were subjected to one million fatigue loading cycles and a sinusoidal wave load was repeated at frequency of 5 Hz. Note that, since the test program presented in this paper aimed for the preliminary tests of actual-sized beams, one million cycles was selected, which is 50% of two million cycles for the actual-sized beams [29,30]. In general, the prestressed concrete beams in service do not allow cracking. The maximum applied load, used for the cycled loading, *P_max_*, was selected to be equivalent to 60% of the cracking load for each specimen. The minimum load is equal to 1.0 kN equivalent to 10% of selected maximum load in order to avoid impact loads during cycling. The fatigue loading was periodically stopped at 1, 10^3^, 10^4^, 10^5^, 3 × 10^5^, 5 × 10^5^, and 10^6^ cycles, in order to conduct intermediate static tests to monitor the damage accumulation and its effect on the stiffness of beams. The maximum load of intermediate static tests was limited to the peak magnitude of the fatigue load. After completing one million cycles, the beams were tested monotonically, up to the failure to evaluate the fatigue capacity of the prestressed concrete beams. During the fatigue and static tests, crack propagation patterns were monitored and marked on the beam surface.

## 3. Results and Discussions

This section presents test results of each beam, considered in the experimental program, and summarizes the relevant research findings. In the first phase, three beams were tested under fatigue loadings at different load ranges, based on a percentage of the cracking load. Three beams in the second phase were tested under monotonic loading conditions up to a failure for the reference. An overall summary of the tests results is given in Table 3. A full discussions of the results is presented in the following section.

### 3.1. F1FU/S1FU Specimens

**Figure 5:**
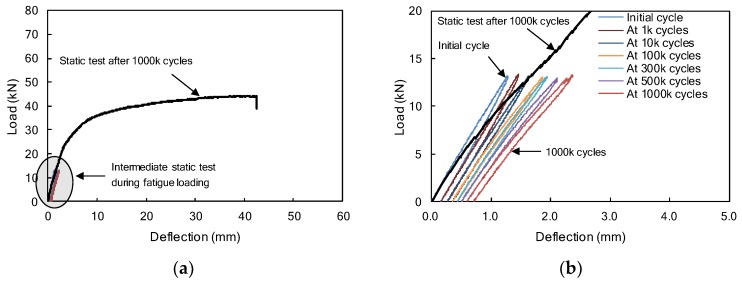
(**a**) Load-deflection curves of F1FU specimens during repeated loading test including static test results after one million cycles. (**b**) A detailed view of the first initial deflection 2.5 mm.

Load-deflection diagrams of the F1FU specimen are presented in Figure 5. The F1FU specimen was post-tensioned with a fully unbonded CFRP bar. The fatigue test was conducted at 5 Hz with a load range between 1.0 kN and 13 kN. As the number of cycles increases up to one million, maximum deflections measured at every intermediate static test increase to 2.36 mm, as shown in Figure 5b. The deflection was measured as 0.70 mm after one million cycles. During fatigue loading, the initial crack appeared on the bottom surface at the mid-span, where the load was applied at approximately 100,000 cycles. The crack developed and propagated toward the compression zone, up to 160 mm from the bottom fiber at one million cycles. 

**Figure 6:**
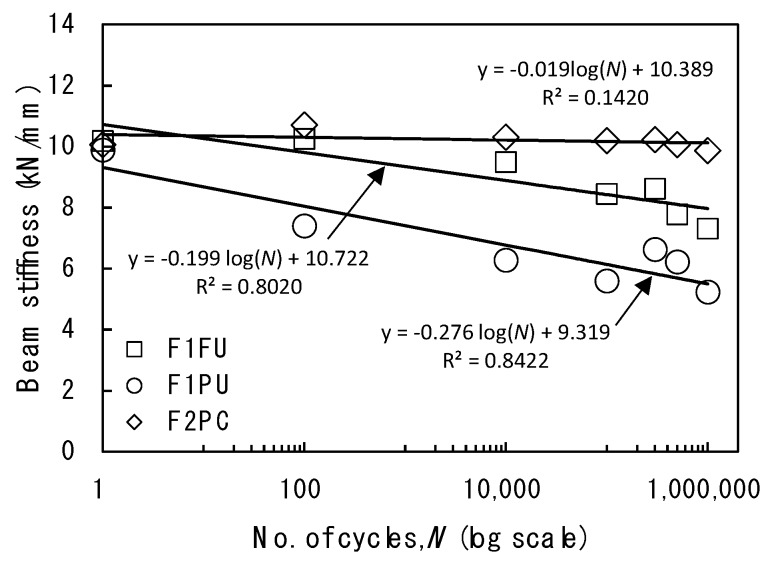
Beam stiffness at various number of cycles.

After the initial cracks were observed in the F1FU specimen, the beam stiffness that was evaluated by the slope of the load-deflection relationship was reduced with the increasing number of cycles, as shown in Figure 6. The stiffness results, shown in Figure 6, are supported by Grace and Sayed (1998, 2000) and Mertol et al. (2006), who observed that the stiffness of prestressed concrete members are degraded under fatigue loading [31,32,33]. It can be attributed to a reduction in the effective moment of inertial, as existing cracks propagate and new cracks develop. Therefore, this reduction in stiffness could be mainly due to the excessive cracking of concrete. Despite beam stiffness degradation during fatigue loading period, the beam was recovered and the cracks closed after one million fatigue loadings.

**Figure 7:**
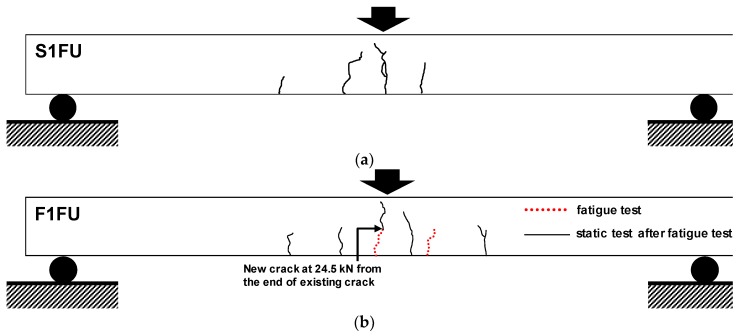
Crack patterns at failure during monotonic loading: (**a**) S1FU specimen (no fatigue loading) and (**b**) F1FU specimen.

**Figure 8:**
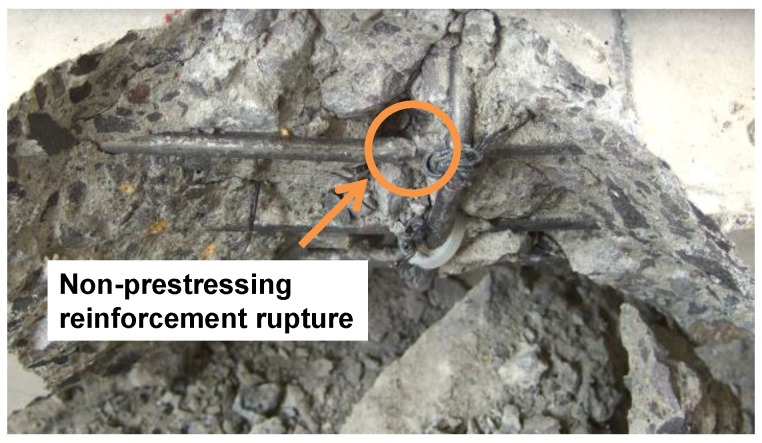
Non-prestressing reinforcement rupture.

**Figure 9:**
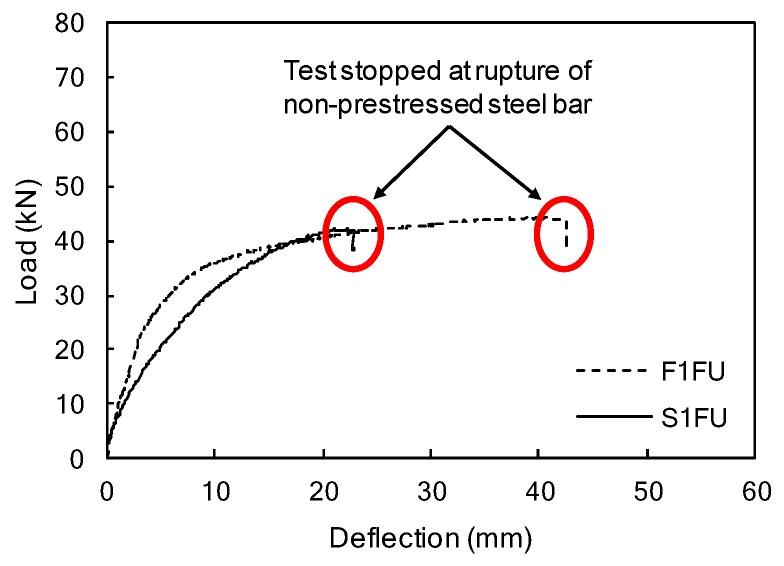
Mid-span load-deflection curves of the F1FU/S1FU specimens.

The F1FU specimen survived one million cycles and was subsequently loaded monotonically to failure. New cracks during monotonic loading appeared at the end of the pre-existing crack, located 100 mm from the mid-span at the load of 24.5 kN, as shown in Figure 7b. The stiffness decreased after the new crack, and maintained its reduced slope in load-deflection curve, with further increase in the load until rupture of non-prestressed steel bar (see Figure 8 and Figure 9). It can be seen that the presence of significant stress concentration, at non-prestressing reinforcement, led to the fracture of the steel bar. It is worth mentioning that most cracks were dictated by the spacing of the shear stirrups. This indicates that the rupture of the steel bar occurs in the vicinity of a stirrup leg at a crack location (see Figure 8); flexural cracks produced high stress levels in the steel bars at crack locations. Like the F1FU specimen, the S1FU specimen also failed by non-prestressing reinforcement rupture as the applied load abruptly dropped (see Figure 9), which can be ascribed to stress concentrations on non-prestressing auxiliary reinforcement.

The non-prestressing reinforcement rupture was observed on both specimens (F1FU/S1FU) under monotonic loading despite exceeding the minimum non-prestressing reinforcements (*A_s_*/*A_s,min_* = 1.13 where *A_s_* is the area of non-prestressed reinforcement). Unbonded FRP bars can improve ductility by relieving the strain from critical sections or averaging strains along unbonded length of bars [7]. However, a small amount of non-prestressed reinforcement can reduce the beneficial effect of unbonded bars in terms of ductility. Therefore, when designing prestressed concrete structures using fully unbonded CFRP bars, an adequate amount of non-prestressing reinforcement should be considered to prevent the unexpected rupture of non-prestressed steel bars. This concept is supported by Lau and Pam (2010), who observed that a higher degree of steel reinforcement in the beam specimen resulted in a more ductile FRP-reinforced concrete [34], and by Qu et al. (2009), who observed that hybrid FRP/steel-reinforced concrete beams, with appropriate reinforcement ratios, exhibited good ductility and load carrying capacity [35].

### 3.2. F1PU/S1PU Specimens

**Figure 10:**
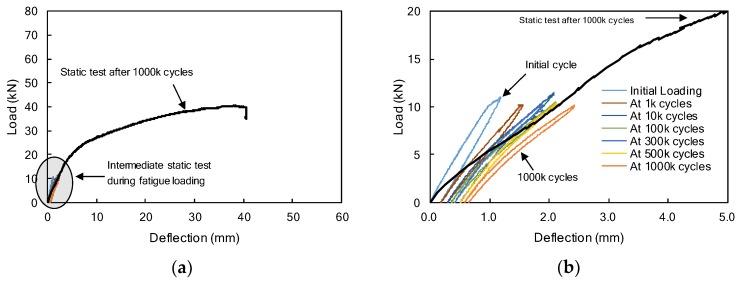
(**a**) Load-deflection curves of F1PU specimens during repeated loading test including static test results after one million cycles. (**b**) A detailed view of the first initial deflection 2.5 mm.

The F1PU/S1PU specimens, as presented in Figure 1b, had the same layout of reinforcements as F1FU/S1FU. However, unlike F1FU/S1FU, a CFRP bar was partially bonded to concrete; 1400 mm unbonded length was provided near the mid-span of the concrete beam by means of a plastic tube. One million fatigue loadings were applied at 5 Hz between 1.0 kN and 10 kN. The maximum deflections, which were measured at every intermediate static test during the fatigue loading test, are presented in Figure 10, including the load-deflection curve of the F1PU specimen at the static test conducted after one million fatigue loadings. The maximum deflections increased up to 2.42 mm at one million cycles (Figure 10b). 

**Figure 11:**
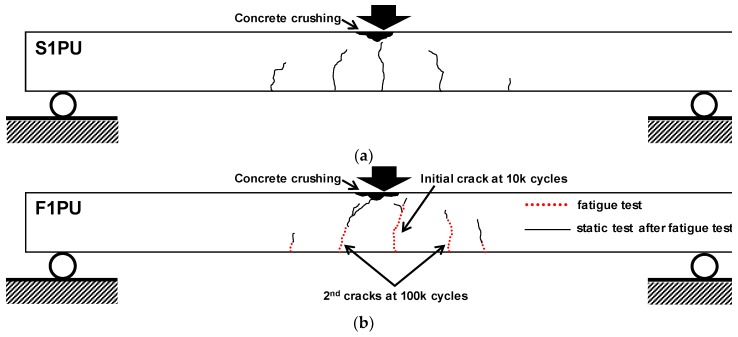
Crack patterns at failure during monotonic loading: (**a**) S1PU specimen (no fatigue loading) and (**b**) F1PU specimen.

The deflection was measured as 0.64 mm after the conclusion of one million fatigue loadings. An initial crack was observed at 10,000 cycles and second cracks appeared at nearly 150 mm distance from the loading point at 100,000 cycles, as shown in Figure 11b. The crack below the loading point propagated toward the compression zone nearly 50 mm from the top fiber after one million cycles. Cracks appearing in the vicinity of 150 mm distance from the loading point extended to nearly 100 mm to 125 mm from the bottom fiber after one million fatigue loadings.

The beam stiffness changed after 1000 cycles, and then, the beam stiffness reduced more rapidly, compared to F1FU with an increasing number of cycles, as shown in Figure 6. This reduction in stiffness is mainly attributable to the excessive crack development toward the compression zone. The cracks were first observed during the intermediate static test, followed by 10,000 cycles. The cracks finally propagated toward 10 mm below the top fiber of the beam during the static test after conclusion of fatigue loading. However, the cracks closed after removing the fatigue load without beam failure occurring after one million fatigue loadings.

**Figure 12:**
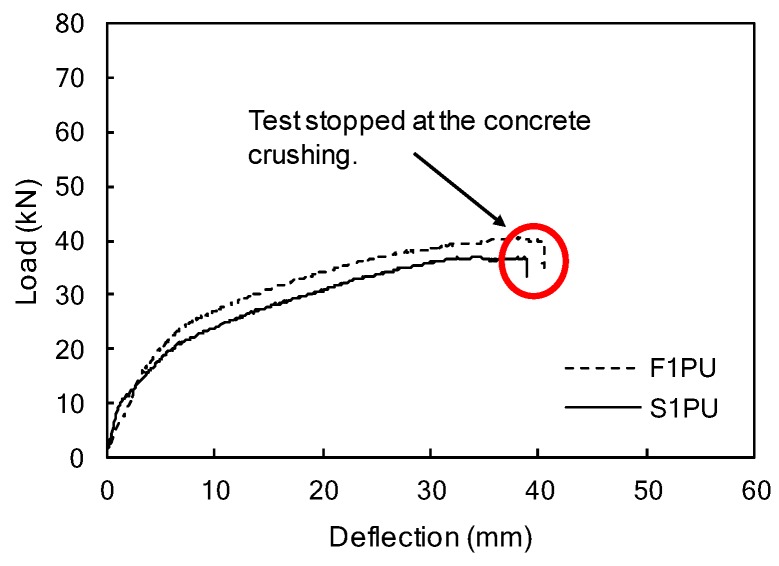
Mid-span load-deflection curves of the F1PU/S1PU specimens.

After the fatigue test, monotonic loading was applied to the F1PU. Figure 12 illustrates tests results for the F1PU specimen including the S1PU specimen under monotonic loading. As the applied load to the F1PU increased up to 15.6 kN, pre-existing cracks reopened, maintaining reduced stiffness, and new cracks started to appear and extend from the end of the pre-existing cracks. As the new cracks began to appear, and developed upon a further increase of the load, the beam stiffness gradually reduced until the F1PU specimen failed in flexure with the crushing of concrete in compression, as shown in Figure 11b. 

Similar flexural behavior and the failure mode were observed for the S1PU specimen when compared to F1PU (see Figure 11 and Figure 12). The deflection of the S1PU specimen linearly increased with the applied load before initial flexural cracking at 9.8 kN. The stiffness was reduced after the initial cracks and gradually kept decreasing with further loading until flexural failure with concrete crushing in the top fiber of concrete beam. Comparing the load-deflection curves of F1PU to S1PU, no significant effect of cyclic loading was observed on the flexural behaviors. This implies that a partially bonded system exhibits a good fatigue performance. Although several cracks appeared and developed toward the concrete compression zone during the fatigue loading period, the flexural response of the F1PU specimen, after repeated loading conditions, is similar to that of the S1PU specimen with same reinforcement layout, which did not undergo fatigue loadings. The ductility of the partially bonded system is evaluated and discussed in Section 3.4 in more detail.

### 3.3. F2PC/S2PC Specimens

**Figure 13:**
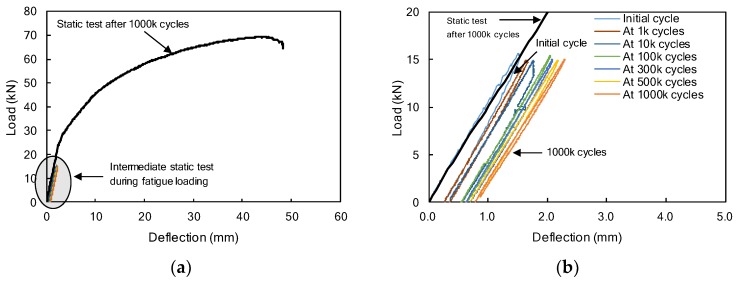
(**a**) Load-deflection curves of F2PC specimens during repeated loading test including static test results after one million cycles. (**b**) A detailed view of the first initial deflection 2.5 mm.

The F2PC/ S2PC specimens were prestressed with two partially bonded CFRP bars and confined with transverse reinforcements, as shown in Figure 1c. A fatigue test, with a frequency of 5 Hz, was conducted on the F2PC specimen and the applied load was varied between 1.0 kN and 15 kN. During the fatigue test, the maximum deflections, were measured at every intermediate static test. and found to have increased to 2.29 mm, as shown in Figure 13b. The deflection was measured as 0.89 mm after one million cycles. 

No flexural cracks occurred during one million fatigue loadings. The bond failure of CFRP bars at the end of the beam were not observed after one million fatigue loadings. A perfect elastic response, corresponding to the initial stiffness, was observed after one million cycles, as shown in Figure 13b. The stiffness of the beam, based on the slope of the load-deflection relationship, is almost identical at the initial loading and reloading, as shown in Figure 13b. This indicates that there was no loss of the beam stiffness due to the fatigue loadings, and this is ascribed to the over-reinforcement (*ρ*/*ρ_b_* = 3.2) and transverse confinement in the compression zone. 

Over-reinforcement and transverse confinement have been proposed as methods to improve the ductility of FRP systems. The absence of plasticity in FRP materials points to the under-reinforced flexural sections undergoing a sudden tensile rupture. Thus, the concrete crushing failure mode of an over-reinforced member is more desirable, due to enhanced energy absorption and greater ductility, leading to a more gradual failure mode [36]. For over-reinforced members, transverse confinement in the compression zone will increase the strain capacity of concrete, and thus, the concrete ductility, since the presence of transverse reinforcement inhibits the propagation of internal cracks [37]. A combination of the two means (over-reinforcement and transverse confinement) is effective in maintaining stiffness during fatigue loading in elastic states, even if over-reinforcement in combination with transverse confinement, was introduced as an important way to induce inelastic behavior in the ultimate stage.

**Figure 14:**
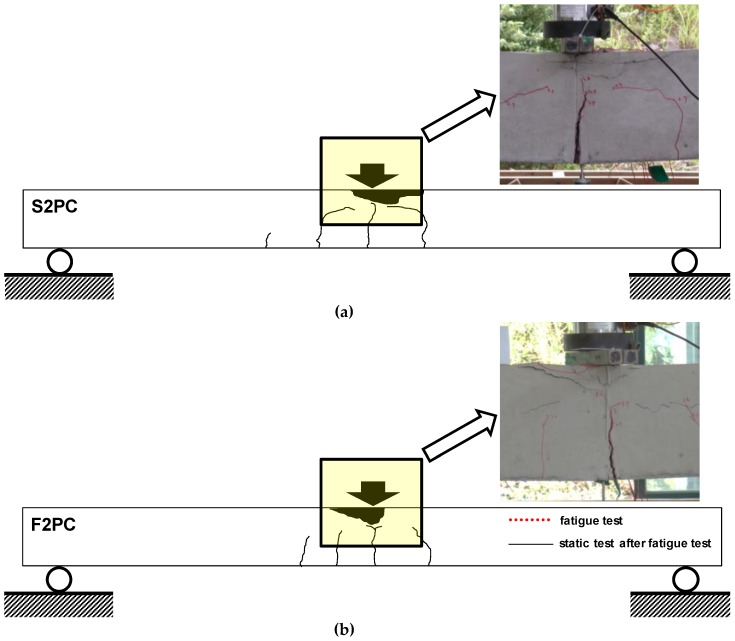
Crack patterns at failure during monotonic loading: (**a**) S1PU specimen (no fatigue loading) and (**b**) F1PU specimen.

**Figure 15:**
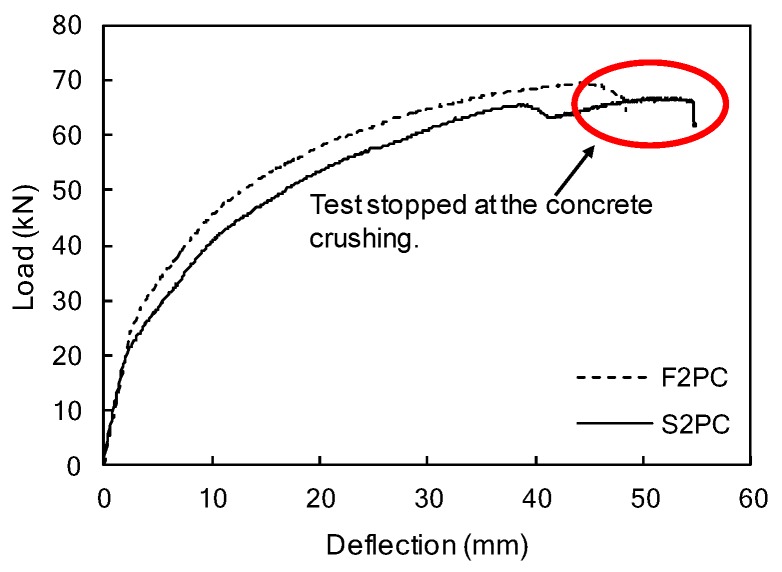
Mid-span load-deflection curves of the F2PC/S2PC specimens.

The static load test of the F2PC specimen followed by the fatigue test was performed to identify the remaining strength after the fatigue loading and the results were compared to a S2PC beam, as presented in Figure 14 and Figure 15. Both F2PC and S2PC specimens behaved linearly up to initial cracks at 24.5 kN, and 18.6 kN, respectively. For the F2PC specimen, when the initial crack occurred at 24.5 kN, a reduction in beam stiffness occurred, and then more cracks formed and propagated toward the loading point with further loading. Finally, the beam failed by concrete crushing at the top surface of the beam, as shown in Figure 14b. 

Compared to the extent of concrete crushing of the F2PC/ S2PC specimens to other specimens, a larger concrete compressive destruction zone of the F2PC/ S2PC specimens are observed (See detail photographs in Figure 14). It can be explained that increasing the ratio of tensile reinforcement (*ρ*/*ρ_b_* = 3.2 for F2PC/ S2PC specimens and *ρ*/*ρ_b_* = 1.6 for others) will shift the location of the neutral axis in the direction of the tension area, so that the extent of concrete used becomes bigger. In other words, when concrete strength increases, the degree of crushing decreases. When it comes to ductility, the neutral axis’ depth of the beam in failure can determine its ductility. Therefore, further study of the practical rule in limiting the neutral axis’ depth seems to be required on high-performance concrete beams to assure adequate ductility levels.

The load-deflection curves of S2PC (no fatigue loading) and F2PC specimens are presented in Figure 15. Consistent with the results of F1PC and S1PC, the static test results showed that the F2PC specimen exhibited similar beam strength to S2PC. The deflection of the S2PC specimen linearly increased with the applied load, before the initial flexural cracking. During one million cycles, neither bond failure of CFRP bars, nor cracks were observed in the F2PC specimen that was prestressed with partially bonded/over-reinforced CFRP bars, and confined with transverse reinforcements. After the completion of one million cycles, the F2PC specimen exhibited similar behavior to the S2PC specimen (no fatigue loading) under monotonic loading (see Figure 15). The initial beam stiffness of both specimens were similar to each other until the first cracking. The flexural behaviors in the post-cracking phase of both F2PC and S2PC exhibited gradual stiffness degradation until failure. The failure modes of both beams were flexural failures with concrete crushing. In spite of one million fatigue loadings, there was no reduction in the load-carrying capacity or beam stiffness observed in the F2PC specimen. Accordingly, this indicates excellent fatigue performance of the F2PC specimen designed with partially bonded bars, an over-reinforced section, and web confinement in the compression zone.

### 3.4. Energy-Based Ductility Index

**Figure 16:**
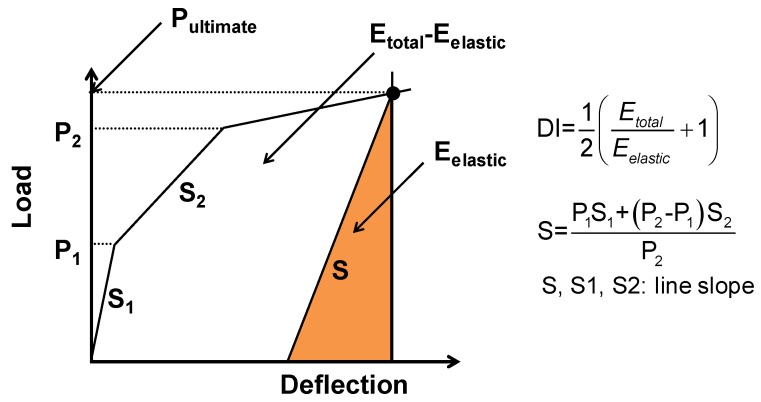
Definition of Ductility Index (DI) [38].

The ductility is defined by the ability to sustain inelastic deformation without loss in the load carrying capacity prior to failure. Conventional ductility is defined as the ratio of deflection at the ultimate load to deflection at yield. This definition cannot be used to evaluate the ductility of concrete members prestressed with CFRP bars, since CFRP bars exhibit linearly elastic behavior until failure. Instead, the ductility of prestressed beams, using CFRP bars, can be calculated by the ratio between elastic and inelastic energies, consumed under the load-deflection curve [32,38,39]. In this study, the Ductility Index (DI) of the tested beams was evaluated on the basis of the energy ratio up to peak load [32],

(2) $$DI=\frac{E_{total}-E_{elastic}}{E_{total}}$$

where *E_total_* is the total energy, calculated as the area under the load-deflection curve up to the peak load, and *E_elastic_* is the elastic energy, which is illustrated in Figure 16. The elastic energy (*E_elastic_*) can be estimated from unloading tests. If unloading data are not available, it can be evaluated using the area of the triangle formed at failure load by the weighted average slope of the two initial straight lines of the load-deflection diagram, as shown in Figure 16 [38]. If the beam is perfectly elastic, DI is 0.0, and perfectly plastic, DI is 1.0. In this study, the DI values were obtained with the weighted average slope in load-deflection curves according to Figure 16.

**Figure 17:**
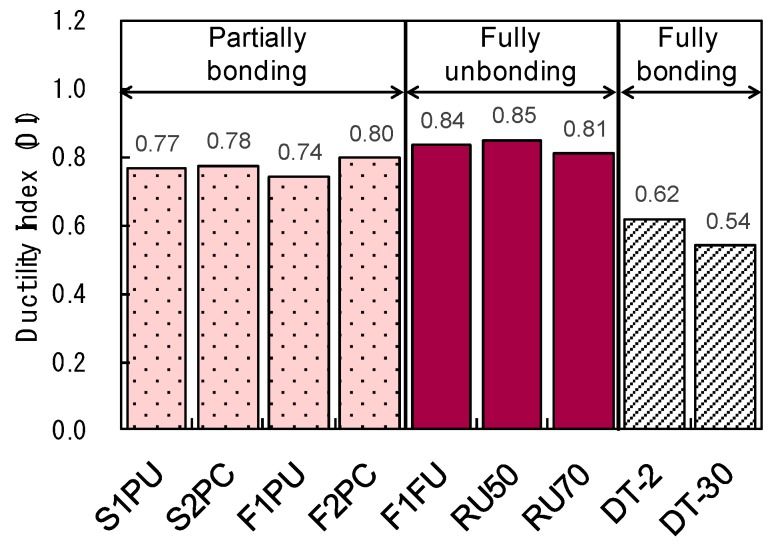
Comparison of ductility index [2,32].

The DI of five beams was evaluated using Equation 2, and the results are presented in Table 3. For comparison purposes, Figure 17 includes the ductility index of concrete beams, prestressed with fully unbonded and bonded CFRP tendons, which was extracted from the literature [2,32]. In Figure 17, the RU50 and RU70 tested beams were under-reinforced with fully unbonded CFRP tendons [2], and the DT-2 and DT-30 specimens used fully bonded CFRP prestressing bars [32]. The details of specimens can be found in the references [2,32]. 

It was found that DI values vary according to bonding conditions - ranging between 0.54 and 0.85 - and it was shown that concrete beams prestressed with fully unbonded/partially bonded CFRP bars have a higher DI than those of specimens prestressed with fully bonded CFRP bars. In general, DI values range from 0.1 to 0.5 for the concrete beams prestressed with fully bonded CFRP tendons [2,38]. The improved DI was observed for concrete beams prestressed with partially bonded CFRP bars, when compared to prestressed beams using fully bonded CFRP bars (see Figure 17). The calculated DI were in the range of 0.74 to 0.80 for these beams, which were prestressed with partially bonded CFRP bars. These values were less than those of concrete beams prestressed with fully unbonded CFRP bars (0.81 to 0.85), but as much as those of beams prestressed with steel bars (0.7 to 0.85 in general). Considering concrete beams prestressed with fully bonded CFRP bars, the DI values of beams with partially bonded CFRP tendons are much higher (0.1 to 0.5 in general). Especially, the effects of over-reinforcement and web-confinement on ductility enhancement in a partially bonded system (e.g., S2PC and F2PC in this study) are shown by making comparisons with S1PU and F1PU specimens, revealing slightly increased ductility regardless of the application of fatigue loadings.

## 4. Results and discussions

Six simply supported beams were tested to study the fatigue behavior and failure character of concrete beams, prestressed with fully unbonded/partially bonded CFRP bars. The experimental program was conducted utilizing fully unbonded/partially bonded bars, web confinement, and over-reinforcement as experimental parameters. Ductility indices of specimens were also evaluated. Based on the experimental program, the following conclusions can be drawn:

1. The F1FU specimen post-tensioned with a fully unbonded bar exhibited good fatigue performance and high ductility. No considerable reduction in beam stiffness or slippage in anchorage was observed during one million cycles. In a static test, conducted after completing one million cycles, the F1FU specimen, despite beam failure by non-prestressing reinforcement rupture, had the highest ductility index.
2. It was observed that S1FU and F1FU specimens experienced failure by non-prestressed reinforcement during static tests. From a design point view, an adequate amount of non-prestressing reinforcement should be considered to prevent abrupt failure due to the unexpected rupture of non-prestressed steel bars.
3. The F1FU/S1FU specimens prestressed with fully unbonded bars failed by non-prestressing reinforcement rupture during monotonic loading. Fully unbonded bars can improve the ductility of prestressed concrete beams, despite the prestressing reinforcement ratio being less than the balanced prestressing reinforcement ratio. However, from a design point, an adequate amount of non-prestressing reinforcement is essential for preventing the unexpected rupture of non-prestressing reinforcement, and to ensure the inelastic behavior of prestressed concrete beams with fully unbonded bars.
4. The over-reinforced and web-confined concrete beams, prestressed by partially bonded bars, i.e., the F2PC specimen, exhibited good fatigue performance with no cracks and no reduction in beam stiffness during one million cycles. In addition, it was found that no reduction in the load-carrying capacity and beam stiffness occurred in a static test, conducted after the conclusion of one million cycles.
5. Improved ductility was observed for beams prestressed with fully partially bonded CFRP bars, when compared with prestressed beams that have fully bonded CFRP bars. Over-reinforced and web-confined concrete beams, prestressed with partially bonded CFRP bars, were proposed, and evaluated to possess good fatigue capacity and durability based on the experimental results.

## Figures and Tables

**Table 1:** Details of test specimens.

Specimen ID	Test Type	Prestressing Reinforcement Ratio (*ρ*/*ρ_b_*)	Initial Prestressing	Unbonded Length (mm)	Web Confinement (mm)
F1FU	Fatigue test	1.6	0.33 *f_pu_*	Fully unbonded	N/A^1^
F1PU		1.6	0.33 *f_pu_*	1400	N/A
F2PC		3.2	0.35 *f_pu_*	1400	1300 (D6@50)
S1FU	Static test	1.6	0.33 *f_pu_*	Fully unbonded	N/A
S1PU		1.6	0.33 *f_pu_*	1400	N/A
S2PC		3.2	0.35 *f_pu_*	1400	1300 (D6@50)

^1^ N/A: Not applicable.

**Table 2:** Measured material properties of carbon fiber reinforced polymer (CFRP) bar and steel reinforcement. (Provided by the manufacturers)

Material	Diameter (mm)	Effective Area (mm^2^)	Yield Strength (MPa)	Ultimate Tensile Strength (MPa)	Ultimate Strain (%)	Modulus of Elasticity (GPa)
CFRP	9.5	70.9	-	2500	1.83	136
Mild steel	6	28.3	400	710	-	200

**Table 3:** Summary of test program and results of fatigue and static tests.

Test Type	Specimen ID	During Repeated Loading			During Monotonic Loading after One Million Cycles			Ductility Index (DI)
		Applied Load (kN)		No. of Cycles at Initial Crack	Load at Initial Crack (kN)	Ultimate Load (kN)	Failure Mode ^2^	
		Min.	Max.					
Fatigue test	F1FU	1.0	13	100,000	24.5	44.3	R	0.84
	F1PU	1.0	10	10,000	15.6	40.5	C	0.74
	F2PC	1.0	15	No cracks	24.5	69.5	C	0.80
Static test	S1FU	N/A ^1^		N/A	20.6	42.3	R	PF ^3^
	S1PU	N/A		N/A	9.8	37.1	C	0.77
	S2PC	N/A		N/A	18.6	67.0	C	0.78

^1^ N/A: Not applicable; ^2^ R: Rupture of non-prestressing reinforcement C: Concrete crushing; ^3^ PF: Premature failure prior to yielding.
